# Estimated gray matter volume rapidly changes after a short motor task

**DOI:** 10.1093/cercor/bhab488

**Published:** 2022-02-08

**Authors:** Gaia Olivo, Martin Lövdén, Amirhossein Manzouri, Laura Terlau, Bo Jenner, Arian Jafari, Sven Petersson, Tie-Qiang Li, Håkan Fischer, Kristoffer N T Månsson

**Affiliations:** Department of Psychology, University of Gothenburg, SE-40530, Gothenburg, Sweden; Aging Research Center (ARC), Department of Neurobiology, Care Sciences and Society, Karolinska Institutet, SE-17177, Stockholm, Sweden; Department of Psychology, University of Gothenburg, SE-40530, Gothenburg, Sweden; Aging Research Center (ARC), Department of Neurobiology, Care Sciences and Society, Karolinska Institutet, SE-17177, Stockholm, Sweden; Department of Psychology, Stockholm University, SE-10691, Stockholm, Sweden; Centre for Psychiatry Research, Department of Clinical Neuroscience, Karolinska Institutet, SE-11364, Stockholm, Sweden; Center for Lifespan Psychology, Max Planck Institute for Human Development, D-14195, Berlin, Germany; Max Planck UCL Centre for Computational Psychiatry and Ageing Research, D-14195, Berlin, London; Centre for Psychiatry Research, Department of Clinical Neuroscience, Karolinska Institutet, SE-11364, Stockholm, Sweden; Centre for Psychiatry Research, Department of Clinical Neuroscience, Karolinska Institutet, SE-11364, Stockholm, Sweden; Department of Medical Radiation and Nuclear Medicine, Karolinska University Hospital, Huddinge S-14186, Stockholm, Sweden; Department of Clinical Science, Intervention and Technology, Karolinska Institutet, SE-14152, Stockholm, Sweden; Department of Medical Radiation and Nuclear Medicine, Karolinska University Hospital, Huddinge S-14186, Stockholm, Sweden; Department of Clinical Science, Intervention and Technology, Karolinska Institutet, SE-14152, Stockholm, Sweden; Department of Psychology, Stockholm University, SE-10691, Stockholm, Sweden; Stockholm University Brain Imaging Centre, SE-10691, Stockholm, Sweden; Department of Psychology, Stockholm University, SE-10691, Stockholm, Sweden; Centre for Psychiatry Research, Department of Clinical Neuroscience, Karolinska Institutet, SE-11364, Stockholm, Sweden; Department of Psychological and Brain Sciences, Dartmouth College, Hanover, NH, US-03755, USA

**Keywords:** finger tapping, motor training, MRI, plasticity, skill learning

## Abstract

Skill learning induces changes in estimates of gray matter volume (GMV) in the human brain, commonly detectable with magnetic resonance imaging (MRI). Rapid changes in GMV estimates while executing tasks may however confound between- and within-subject differences. Fluctuations in arterial blood flow are proposed to underlie this apparent task-related tissue plasticity. To test this hypothesis, we acquired multiple repetitions of structural T_1_-weighted and functional blood-oxygen level-dependent (BOLD) MRI measurements from 51 subjects performing a finger-tapping task (FTT; á 2 min) repeatedly for 30–60 min. Estimated GMV was decreased in motor regions during FTT compared with rest. Motor-related BOLD signal changes did not overlap nor correlate with GMV changes. Nearly simultaneous BOLD signals cannot fully explain task-induced changes in T_1_-weighted images. These sensitive and behavior-related GMV changes pose serious questions to reproducibility across studies, and morphological investigations during skill learning can also open new avenues on how to study rapid brain plasticity.

Skill learning can induce morphological modifications in the human brain over the course of training (Draganski et al. 2004; Wenger et al. 2017). Several weeks were first deemed necessary for neuroplastic changes to occur, but mounting evidence suggests that changes in measures of brain morphology may be detectable already after a few days (Kwok et al. 2011; Irmen et al. 2020) or even within minutes (Månsson et al. 2020). For example, cortical thickness of the motor cortex linearly increases during 1 h of balance training in healthy adults (Taubert et al. 2016). More recently, rapid increases in estimates of gray matter volume (GMV) and cortical thickness in the human visual cortex were reported to occur already after a few minutes of passively viewing visual stimuli during T_1_-weighted magnetic resonance imaging (MRI) acquisitions (Månsson et al. 2020). Thus, commonly used measures of brain’s morphology seem more sensitive than previously believed.

The nature of these apparent tissue changes is, however, debated. Transient stimulus-dependent increases in cortical GMV may stem from astrocytes swelling, spines and synapses production, followed by spines maturation and pruning (Xu et al. 2009; May 2011; Donato et al. 2013; Moczulska et al. 2013; Kuhlman et al. 2014). Alternatively, T_1_-weighted signal changes may be ascribed to task-related changes in blood flow. Arterial blood has higher T_1_ (spin–lattice relaxation time) than brain tissues, therefore having the potential to induce apparent tissue changes in T_1_ images (Ge et al. 2017). Investigation of brain activity with functional MRI (fMRI) have shown that changes in the blood-oxygen level-dependent (BOLD) signal, reflective of fluctuations in both blood flow and oxygenation, occur early during motor-skill learning (Lefebvre et al. 2015). Dynamic changes in functional brain architecture are induced rapidly in the cerebellum, striatum, and other motor-related cortical areas (Ungerleider et al. 2002; Park et al. 2010), followed by a more slowly evolving reorganization of the primary motor areas during motor-skill learning (Karni et al. 1995; Ungerleider et al. 2002). Thus, not only motor skills, but also BOLD signal and T_1_-weighted estimates of GMV can rapidly change. However, these measures are typically investigated independent from each other, and multimodal plastic changes induced by a motor-skill learning task have not yet been investigated.

The implications of immediate task-effects on T_1_-weighted signal is, per se, not a trivial issue in MR research, regardless of the biological mechanisms sustaining rapid changes in estimates of GMV. Research MRI protocols often include structural and task-fMRI sequences in variable order within the same scanning session. It is also common practice during long scanning sessions to ease tension and make the experience more bearable by offering the participants music or movies during the acquisition of structural sequences. The impact of these activities on T_1_-weighted GMV measures has long been underestimated, potentially leading to reliability issues in longitudinal study designs, as well as to reproducibility issues across studies.

The aim of this study is threefold: (1) to explore whether changes in GMV estimates based on T_1_-weighted imaging can be induced by task execution (i.e., 2-minute execution of a finger-tapping task, FTT) (RQ_1_) and whether these motor-related changes would show regional overlap with task-related BOLD signal (RQ_2_); (2) to determine the evolution of changes in T_1_-based estimates of GMV (RQ_3_) and BOLD signal (RQ_4_) over 12 and 26 min of training on the FTT; (3) to test the relationship between BOLD signal fluctuations and GMV estimates variation over time (RQ_5_). To this aim, we acquired 6 images of repeated T_1_-weighted MRI measurements and 4 images of fMRI measurements from 51 subjects performing FTT with alternating 2 min of rest over 30-minute continuous scanning (Fig. 1). About half of the participants (*n* = 27) continued performing the task for another 30 min and underwent a total of 12 acquisitions of T_1_-weighted and 8 acquisitions of BOLD-fMRI measurements (Fig. 1).

**Figure 1:**
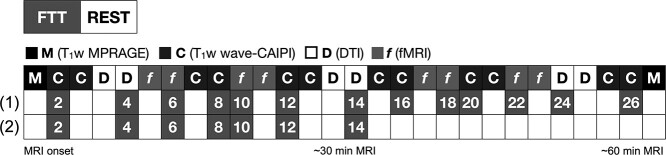
Protocol description. The figure summarizes the experimental session. For 30 min, all subjects underwent FTT, alternating with resting, each acquisition lasting 2 min (in total 14 min of FTT during ~30-minute scanning). This first half of the protocol consisted of 6 acquisitions with wave-CAIPI T1 weighted images (“C”) and 4 acquisitions with BOLD fMRI (“*f*”). In the second half of the protocol, 27 subjects (1) continued performing FTT (with alternating resting) for another 30 min (in total 26 min of FTT during ~60-minute scanning), whereas 24 subjects (2) rested for 30 min. In total, we acquired 12 images with wave-CAIPI T1 weighted brain scans, and 8 images with BOLD fMRI. In addition, two T1 weighted MPRAGE images (“M”) were acquired in the beginning and at the end of the scanning session. DTI was acquired but data are not included in this manuscript. An alternative figure in color is available at https://github.com/neuronsson. **Abbreviations**: CAIPI, Controlled Aliasing in Parallel Imaging; DTI, diffusion tensor imaging; fMRI, functional magnetic resonance imaging; FTT, finger-tapping task.

In brief, we report rapid changes in estimated GMV during FTT execution compared with rest. These changes were specific to task execution and differed from time-related changes occurring over 12 and 26 min of training. BOLD signal fluctuations did not overlap nor correlate with T1-based GMV change over time, evincing that BOLD signal variations cannot fully explain task-induced T_1_-signal changes. Task-related structural imaging may thus have the potential to advance our knowledge on rapid brain plasticity.

## Material and Methods

### Subjects and Recruitment

The study was approved by the ethics committee of region Stockholm in Sweden (Regionala Etikprövningsnämnden, Dnr: 2018/671-32) and complied with the principles of the Declaration of Helsinki. Demographic information, self-rated health and handedness of the participants are reported in Table S1, Appendix A, Supplementary Information. In brief, 52 healthy, right handed adults (mean age 26.1 ± 3.94 [standard deviation, SD]; 27 females [52.9%] and 25 males [47.1%]) were recruited in 2 waves including 27 and 25 subjects, respectively. The recruitment periods were about 5 weeks apart. The advertisement was posted on bulletin boards at Karolinska Institutet, Södertörn University, and Stockholm University in Stockholm. The advertisement was also shared with student union groups for psychology students at Karolinska Institutet and IT students at Stockholm University. Participants were compensated with a gift card worth 300 SEK. One subject from the second wave was excluded due to technical issues during the scanning procedure, resulting in 27 and 24 subjects included in each wave. Eligibility was assessed with a simple questionnaire regarding MRI safety, handedness (Edinburgh Handedness Inventory, see Table S1) (Oldfield 1971), self-rated health (SRH-7) (Eriksson et al. 2001), physical impairments, playing musical instruments, playing video games, and fluency in Swedish.

### Finger-Tapping Task and Experimental Protocol

A visually paced, complex sequence multifinger-tapping task was designed based on previous studies of motor training (Karni et al. 1995; Walker et al. 2002). The sequential FTT required participants to press 4 differently colored buttons on the input device, using 4 fingers of their nondominant left hand, repeating the 6-number sequence 1 4 2 3 1 3 as quickly and as accurately as possible. This numeric sequence was displayed to the participants at all times to avoid any working memory component to the task. Each acquisition of FTT lasted 2 min, and was alternated with 2 min of rest. Performance on the task was calculated as the number of correctly tapped sequences completed in each 2-minute FTT-run. FTT and rest were pseudorandomized, to ensure that the presentation of FTT/rest and rest/FTT pairs were balanced throughout the MRI scanning for each scanning modality (Fig. 1).

Before enrolment in the study, participants were asked to perform a short computerized screening test. The purpose of this screening test was to exclude participants with high baseline proficiency at the FTT, in order to minimize ceiling effects during the main study. Participants were blinded to the criteria for screening. The screening test had a duration of 1 min and the participants had to repeat a sequence of numbers (3 2 4 1) as fast and as accurately as possible. The screening test was almost identical to the FTT later used in the scanner, the sequence of numbers used for the screening was 2 digits shorter and the FTT inside the scanner took 2 min instead of 1 min. Participant scores (i.e., the total number of sequences correctly performed during 1 min) were standardized with *z*-transformation and participants with a score 1 SD above the group’s average were excluded from the study.

Initial performance was measured once again immediately before the scanning session. This prescan test was identical to the screening test, both with regards to instruction and task design, but a new sequence was used (1 3 4 2) for FTT. Screening and prescan tests were performed on a laptop computer using keys 1–4 for user input. The software script used for displaying information to the participants and registering their input was developed in E-Prime 2.0 (Psychology Software Tools, 2015). Participants were given detailed instruction before entering the scanner, informing them that the task in the scanner would be nearly identical to the previous screening and prescan task, but with a different number sequence. They were instructed that the left index to little fingers were numbered from 1 to 4 and that they were to perform finger-tapping sequences as fast and accurately as possible according to visually presented sequences. The participants were told that they would perform 2 min of finger tapping alternated with 2 min of rest multiple times, and it was emphasized that the goal was to improve in FTT performance as much as possible.

The whole scanning acquisition lasted ~60 min. In the first half of the protocol (~30-minute MRI scanning), all participants performed 2-min of FTT alternated with resting (representing 12 min of FTT in total; Fig. 1). In the second half of the protocol, 27 subjects (study wave 1) kept on alternating between FTT and rest (totaling 26-minute FTT during ~60-minute MRI scanning; Fig. 1). Participants from study wave 2 (*n* = 24) only rested for 30 min during the second half of the protocol (Fig. 1). After each FTT, participants received visual feedback on the number of completed correct sequences. Participants were also informed when the first quarter of the session was completed, and respectively when they reached one half and the last quarter of the session.

### MRI Acquisition

All MRI data acquisition was conducted on a whole-body 3 T clinical MRI scanner (Magnetom Prisma-fit, Siemens Medical Solutions, Erlangen, Germany) at Karolinska hospital in Huddinge with a gradient system of 8 Gauss/cm and a compact 64-channel phased-array receiving head coil. Structural images were acquired with two different T_1_-weighted sequences: MPRAGE (magnetization-prepared rapid acquisition with gradient-echo) and wave-CAIPI MPRAGE (Polak et al. 2018). MPRAGE is the gold-standard sequence to assess brain anatomy and is generally preferred for voxel-based morphometry (VBM) analyses. Conventional MPRAGE sequences were acquired in the resting condition only once before and once after all the training sessions. The wave-CAIPI (Controlled Aliasing In Parallel Imaging) MPRAGE was used to obtain rapid images of the brain during FTT and rest, each lasting 2 min.

The conventional 2-fold GRAPPA accelerated MPRAGE protocol lasted 4:26 min to acquire one timeframe of 3D T_1_-weighted brain volume and used the following main acquisition parameters: TE = 2.52 ms; TR = 1900 ms; TI = 900 ms, flip angle = 9°, 1 mm^3^ isotropic voxel size with matrix size = 256 × 256 × 176. The 9-fold accelerated wave-CAIPI MPRAGE protocol lasted 120 s to acquire one timeframe of T_1_-weighted 3D image volume and used the following parameters: TE = 3.46 ms; TR = 1829 ms; TI = 900 ms, flip angle = 7°, 1 mm^3^ isotropic voxel size with matrix size = 256 × 256 × 192. BOLD contrast images were acquired using the following parameters: TE = 33 ms; TR = 720 ms; flip angle = 52°; field of view: 1872 × 1872 mm^3^, reconstruction matrix size: 104 × 104, and 8-fold accelerated sing-shot echo-planar imaging. Seventy-two slices with a thickness of 2 mm were acquired to capture the whole brain. Diffusion tensor imaging (DTI) acquisitions were also carried out. DTI data are not included in the current manuscript.

### Preprocessing of Structural and Functional Imaging Data

Structural imaging data, acquired with the conventional MPRAGE and wave-CAIPI sequences, were preprocessed with the CAT12 Toolbox (http://www.neuro.uni-jena.de/cat/) running in Statistical Parametric Mapping (SPM) version 12 (https://www.fil.ion.ucl.ac.uk/spm/software/spm12/), by using the longitudinal preprocessing pipeline. The longitudinal pipeline accounts for intrasubject variability and consists in intrasubject realignment, bias correction, segmentation, normalization, and smoothing. Longitudinal images were first registered to the mean image for each subject by an inverse-consistent realignment and segmented into gray matter (GM), white matter (WM) and cerebrospinal fluid probability maps. For each subject, spatial normalization to the Montreal Neurological Institute (MNI) standard space (Mazziotta et al. 2001) was then estimated for the mean image of all timepoints and applied to all images. Smoothing was performed with an 8 mm full width at half-maximum (FWHM) Gaussian kernel.

Functional images were preprocessed with Data Processing Assistant for Resting-State fMRI Advanced (DPARSFA) (http://rfmri.org/DPARSF). Slice-timing was carried out to correct for the temporal offsets between slices. Images were then realigned to correct for head motion, and acquisitions with motion exceeding the threshold of 3 mm were discarded from further analyses. Only 2 sessions were discarded: the first FTT session for one subject, and the third rest session for another subject. Both subjects belonged to the 60-minute training protocol. Functional images were coregistered to the structural images; to this purpose, the first half of the acquisition (4 sessions) were coregistered to the first conventional MPRAGE acquisition, whereas the second half of the acquisition (4 sessions) was coregistered to the second conventional MPRAGE. Structural images were then segmented and normalized to the MNI space using DARTEL (Diffeomorphic Anatomical Registration Through Exponentiated Lie Algebra) (Ashburner 2007) as implemented in DPARSFA. The parameters resulting from the normalization procedure were then applied to the functional images. Functional images were smoothed with an 8 mm FWHM Gaussian kernel.

### Test–Retest Reliability, Quality and Validation of the Wave-CAIPI Sequences

The intraclass correlation coefficient (ICC) (Bartko and Carpenter Jr 1976; Koo and Li 2016) was calculated as a measure of test–retest reliability. ICCs were calculated between the first and the last acquired image in wave 1 and wave 2 separately (see also Fig. 1). Further, ICCs were calculated on rest and FTT-based T1w wave-CAIPI conditions separately. In total, 4 ICCs are reported (i.e., ICCs across the whole-brain and between 2 timepoints). CAT12 provides quality ratings of the T1w images, accounting for noise contrast ratio, inhomogeneity contrast ratio, and root mean square resolution (http://www.neuro.uni-jena.de/cat12-html/cat_methods_QA.html#Dahnke:2016).

Wave-CAIPI MRPAGE has already been proven to be comparable quality as conventional high-resolution MPRAGE acquisitions (Longo et al. 2020). Nonetheless, prior to investigating our primary research questions (RQ), we explored voxel-to-voxel correlations between GMV estimates obtained by the gold-standard MPRAGE and by the wave-CAIPI sequences, respectively. GMV estimates obtained with the wave-CAIPI and MPRAGE acquisitions were significantly correlated, with threshold-free cluster enhancement (TFCE) corrected *P* < 0.001 for a positive correlation in 99.5% of the voxels (98.9% with TFCE-corrected *P* < 0.001). An exhaustive description of the statistical analyses used for this quality check and resulting findings is reported in Appendix C, Supplementary Information and represented in Fig. S3, Appendix C, Supplementary Information. A comparison of two protocols (30 min vs. 60 min) was also carried out on the MPRAGE images (see Appendix D and Table S6, Supplementary Information). The protocol yielded comparable effects on GMV estimates.

### First-Level fMRI Analysis

First-level analyses, corrected for motion parameters, were first carried out on functional images to calculate the FTT versus rest contrast. This contrast (FTT vs. rest) was used in further analyses, to explore the effect of the amount of FTT training on BOLD signal.

### Research Questions

We had three main aims:

1) To explore whether changes in GMV estimates based on T_1_-weighted imaging can be induced during task execution, namely over 2 min of FTT performance (RQ_1_) and whether these motor-related changes would show regional overlap with task-related brain activity (RQ_2_);
2) To determine the evolution of changes in T_1_-based estimates of GMV (RQ_3_) and BOLD signal (RQ_4_) over 12 and 26 min of FTT;
3) To test the relationship between BOLD signal and GMV estimates (RQ_5_).

### Statistical Analysis

#### Effect of FTT and Time on GMV (RQ_1_, RQ_3_)

Two GLM-based flexible factorial designs were used to test for changes in GMV estimates in the shorter (12-minute FTT; *n* = 51) and longer (26-minute FTT; *n* = 27) FTT protocols. To investigate the effects of the short protocol, the model included all participants (*n* = 51), and two within-subject factors were set: condition (FTT and resting) and time (in total 6 images). To explore the effects of the longer FTT protocol we focused on the 27 subjects who were assigned to the 26-minute FTT-training, with additional levels for the factor time (in total 12 images) (Fig. 5*a*). Main effects of condition, time, and condition × time interaction on GMV estimates were tested. The main effect of condition was tested to answer to RQ_1_ (Can changes in estimated GMV be detected during task performance?); the main effect of time was tested to answer to RQ_3_ (How do changes in estimated GMV induced by the execution of FTT, compared with rest, evolve over the course of 12- and 26-minute training?). The condition × time interaction was used to test whether task-induced rapid changes on T_1_-based GMV estimates were dependent on the time spent training. The primary threshold for significance was set at *P* < 0.001, uncorrected; voxels surviving the primary threshold were further corrected for multiple testing with a family-wise error (FWE) rate approach at cluster level, with a threshold of *P* < 0.05. See Appendix F, Supplementary Information, for a more detailed investigation of the task period compared with the following rest period in wave 2.

#### Task and Time Effects on BOLD Signal Changes (RQ_2_, RQ_4_)

First-level contrast (FTT vs. rest) was used for second level analyses. Prior to exploring the effects of time on brain functional activity, a one-sample *t*-test analysis was preliminarily carried out to ensure the existence of brain activity differences between the task and the resting condition in the whole sample (in total 10 min of FTT). The analysis was corrected for total estimated GMV. The primary threshold for significance was set at *P* < 0.001, uncorrected; voxels surviving the primary threshold were further corrected for multiple testing with a FWE rate approach at cluster level, with a threshold of *P* < 0.05.

We then proceeded to investigate the effect of time during the short and long training protocols. A paired *t*-test was used to investigate the effect of the amount of training (10 min compared with 6 min) during the short training protocol. A flexible factorial design was used to focus on the long training protocol, with image acquisitions as a 4-levels within-subject factor (6-, 10-, 18-, 22 min; Fig. 6*a*). The analysis was corrected for estimated GMV. The primary threshold for significance was set at *P* < 0.001, uncorrected; voxels surviving the primary threshold were further corrected for multiple testing with a FWE rate approach at cluster level, with a threshold of *P* < 0.05.

#### Associations between BOLD Signal and T_1_-Based Estimates of GMV (RQ_5_)

To test whether the task-related effects detected in T_1_-based estimates of GMV could be mediated by task-related changes in BOLD signal, voxel-to-voxel correlations between the 2 modalities were tested in FSL (FMRIB Software Library) (Jenkinson et al. 2012). The “randomize” command implemented in FSL (Winkler et al. 2014) allows for the inclusion of voxel-specific covariates in the analysis via the “—vxl” option. We used the wave-CAIPI images to compute the FTT versus rest contrast for each subject, which was then entered as dependent variable in the correlation analysis. The same first-level contrast from the BOLD-fMRI analysis was entered as voxel-specific, independent variable. The analysis was masked for clusters where the task elicited statistically significant activations. FSL provides a nonparametric permutation-based statistics (Smith et al. 2006). The number of permutations was set at 5000. The threshold for significance was set at *P* < 0.05, corrected for multiple comparisons at cluster level with a TFCE approach (Smith and Nichols 2009). A separate, similar analysis was carried out by computing the voxel-wise difference in estimated GMV and functional activity between the first and last acquisition, respectively for each modality, for each subject (~30-minute protocol). Estimated GMV difference were entered as dependent variable, whereas BOLD signal difference was entered as voxel-specific independent variable.

**Figure 2:**
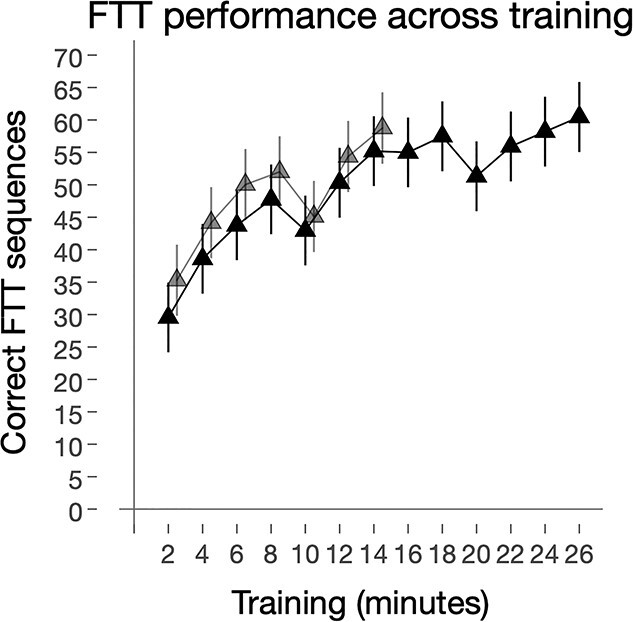
Improvements after FTT training. The total number of correct FTT sequences increases over the course of training. Twenty-seven participants completed in total 13 sessions (26 min) of the FTT and 24 subjects completed 7 sessions (14 min). Black solid line and triangles represent all subjects (*n* = 51), whereas those that only completed 7 training sessions are represented by the light gray line and triangles. All error bars represent 95% CI. **Abbreviations**: FTT, finger-tapping task.

**Figure 3:**
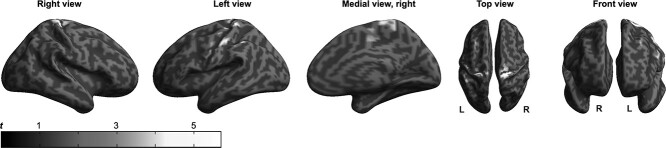
Effects of FTT execution on estimated GMV. The figure shows clusters where statistically significant differences in whole-brain voxel-wise GMV estimates between the 2-minute FTT acquisitions and the 2-minute resting acquisitions were detected over the short (*n* = 51) training protocols. GMV estimates in the depicted clusters (located mainly in the precentral and postcentral gyri) were increased during rest compared with the execution of the FTT. Clusters with *P* < 0.001 at the uncorrected level that further survived FWE-correction for multiple testing at cluster level (*P* < 0.05) are reported. Significant clusters are overlaid to the surface rendering provided by SPM12. An alternative figure in color is available at https://github.com/neuronsson  **Abbreviations**: FTT, finger-tapping task; FWE, family-wise error; GMV, gray matter volume; SPM, statistical parametric mapping.

#### Associations between Motor-Skill Performance, Estimated GMV and BOLD Signal

Stata/MP v.15.1 (https://www.stata.com/) was used to test associations between motor-skill performance and measures of brain response and GMV. FTT performance was defined as the maximum number of correctly completed sequences for each 2-minute FTT-session. Because the largest improvements in motor-skill learning was achieved during the first 7 sessions (~30-minute scanning; *n* = 51), brain-behavior associations were restricted to these timepoints (see also Fig. 2). GMV and BOLD signal changes (FTT vs. rest) at each timepoint were extracted from each cluster (average signal within each cluster) where a significant effect of condition or time was detected.

First, we performed linear regressions on each subject’s FTT performance (time as the independent variable), and the standardized beta value (*β*), representing the slope of FTT performance improvement, were extracted for each individual. Next, we performed linear regressions on each subject’s estimated GMV and BOLD signal change (time as the independent variable), and the standardized beta value (*β*), representing the slope of GMV estimates or BOLD signal variation over time, respectively, were extracted for each individual. Finally, linear regressions were performed, including each subject’s FTT slope (*β*) as a dependent variable and each brain cluster’s slope (*β*) as the independent variable. Further, linear regressions were subject to Monte Carlo permutation testing with 5000 repetitions, and alpha was set a *P* < 0.05. Additionally, voxel-wise correlations between FTT performance improvement (slope) and pre- to post-training differences in estimated GMV and BOLD signal were tested (see Appendix E, Supplementary Information).

## Results

### Behavioral Data

As depicted in Figure 1, all subjects (*n* = 51) performed 7 sessions of FTT the first half of the scanning (~30 min), whereas 27 subjects performed 6 additional FTT sessions in the second half (totaling ~60-minute scanning and 13 FTT sessions). Behavioral performance improved over the course of training (*Z* > 16.8; *P* < 0.001; Fig. 2). Specifically, the number of correctly completed FTT sequences almost doubled from the first (32.2; 95% CI 28.5, 35.9) to the seventh training session (totaling 14 min of FTT training; 56.7; 95% CI 53.0, 60.5), with a within-group Cohen’s *d* effect size of 1.78 (95% CI 1.47, 2.08). Between the 8th and 13th training session a slight increase in behavioral performance was noted (FTT session 13, 59.2; 95% CI 54.0, 64.3; *Z* = 4.02, *P* < 0.001), with an effect size of *d* = 0.19 (95% CI −0.09, 0.46).

### Effects of Task Execution on GMV Estimates and BOLD Signal (RQ_1_, RQ_2_)

#### Can Changes in T_1_-Weighted Estimates of GMV be Induced by Short Bouts of FTT Execution? (RQ_1_)

Statistically significant effects of task (7 acquisitions of 2-minute FTT execution compared with 7 acquisitions of 2-minute rest) were detected in the primary motor and somatosensory cortices, with large overlap between the shorter (*n* = 51) and longer (*n* = 27) protocols. In particular, GMV estimates in bilateral precentral and postcentral gyri were larger during rest compared with FTT (Cohen’s *d* = 0.11 and 0.47 for right and left hemispheres, respectively) (Fig. 3 and Table 1; see Fig. S1, Table S2 in Appendix B, Supplementary Information for a comparison between the shorter and longer protocols). No statistically significant condition × session interaction was detected at either protocol, indicating that the estimated volumetric difference between FTT and rest conditions remained stable over time.

**Table 1:** Effect of FTT execution on gray matter volume

*p* FWE	Ce	*F*	MNI coordinates (maxima)	Direction	GMV location
*Main effect of condition (FTT* vs. *rest), shorter protocol (n = 51)*					
<0.001	2220	36.83	19, −27, 70	Rest > FTT	R PreCG, PostCG
<0.001	1704	33.04	−55, −42, 48	Rest > FTT	L Supramarginal gyrus
<0.001	1201	26.18	−24, −30, 66	Rest > FTT	L PostCG, PreCG
0.004	745	23.15	−24, 22, 51	Rest > FTT	L SFG

**Abbreviations**: Ce, cluster extent; F, *F*-score; FWE, family-wise error; L, left; PreCG, precentral gyrus; PostCG, postcentral gyrus; SFG, superior frontal gyrus; R, right.

#### Are Task-Related Changes in BOLD Signal and GMV Located in the Same Regions? (RQ_2_)

**Figure 4:**
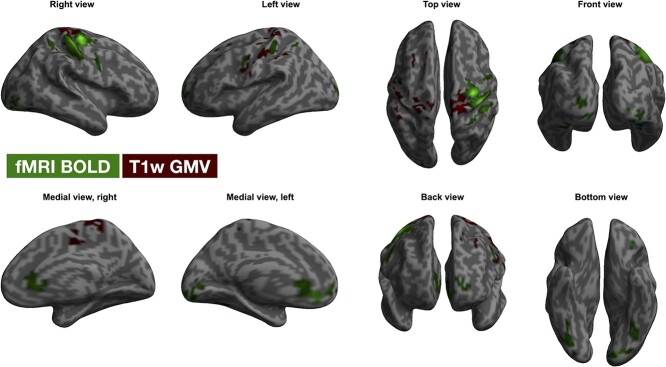
Task-effect on BOLD signal changes and T_1_-based estimates of GMV. The figure shows clusters where statistically significant task-effects were detected at the fMRI analysis (BOLD signal, green clusters) and at the VBM analysis (T_1_-based estimates of GMV, red clusters). The clusters were located in similar regions, despite scarce to no voxel-overlap was evident. Clusters with *P* < 0.001 at the uncorrected level that further survived FWE-correction for multiple testing at cluster level at *P* < 0.05 are reported. Significant clusters are overlaid to the surface rendering provided by SPM12. **Abbreviations**: BOLD, blood-oxygen level-dependent; FWE, family-wise error; GMV, gray matter volume; SPM, statistical parametric mapping; VBM, voxel-based morphometry.

As expected, statistically significant effects of task execution on the BOLD signal were detected on several clusters involved in motor planning and execution, including motor and somatosensory cortices, where activity was higher during FTT execution compared with rest (Fig. S2, Table S3 in Appendix B, Supplementary Information). Although some were adjacent, none of these clusters overlapped with the clusters where statistically significant task-effects on estimated of GMV were detected, either at the uncorrected nor FWE-corrected thresholds (Fig. 4).

### Time-Related Effect of Repeated FTT on Estimated GMV and BOLD Signal (RQ_3,4_)

#### Do Estimates of GMV Change over the Course of 12- and 26 min of FTT Training? (RQ_3_)

**Figure 5:**
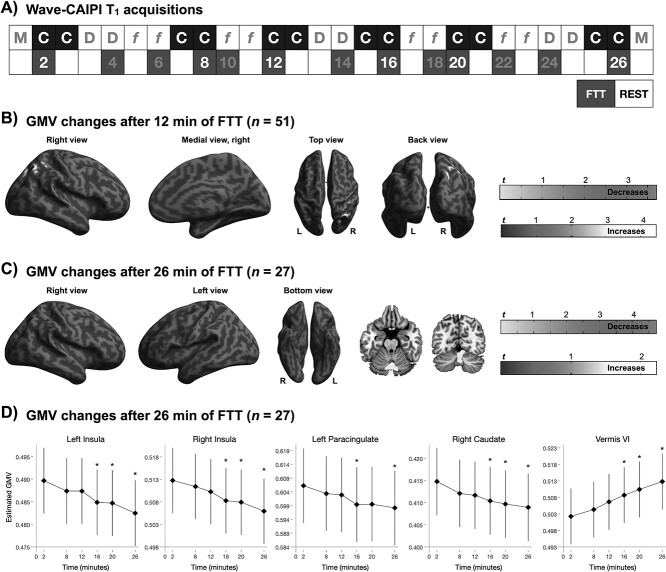
Effects of 12-minute and 26-minute FTT-training on GMV estimates based on T_1_-weighted imaging. (**A**) Structural images of the brain were acquired with a fast 3D T_1_w acquisition (wave-CAPI MPRAGE). Six CAIPI-T_1_ sessions (3 FTT, 3 rest) were acquired on all subjects (*n* = 51), for a total of 12-minute FTT-training. Additional 6 sessions (3 FTT, 3 rest) were acquired from 27 subjects, totaling 26 min of FTT. (**B**) Whole-brain, voxel-wise estimates of GMV after 12 min of FTT training (*n* = 51, 3 acquisitions of FTT/rest) were larger in the left superior occipital lateral cortex, but reduced in the right thalamus and right insula. (**C**) After 26-minute FTT-training (*n* = 27, 6 acquisitions of FTT/rest), a reduction in GMV estimates was observed in the right caudate and paracingulate gyrus. Significant clusters are overlaid to the surface rendering provided by SPM 12. Red clusters represent regions where estimated GMV was increased from pretraining to 12 min of FTT, whereas green clusters represent regions where estimated GMV decreased during training. Clusters with *P* < 0.001 at the uncorrected level that further survived FWE-correction for multiple testing at cluster level (*P* < 0.05) are reported. (**D**) Changes in GMV estimates was approximately linear over 26-minute training, and became statistically significant after 16 min compared with pretraining. Statistical significance is denoted with stars, and all error bars represent the standard error. An alternative figure in color is available at https://github.com/neuronsson  **Abbreviations**: FTT, finger-tapping task; FWE, family-wise error; GMV, gray matter volume; SPM, statistical parametric mapping.

The wave-CAIPI protocol was designed to generate a whole-brain T_1_-weighted image volume in 2 min. Therefore, 6 T_1_-weighted images (3 while executing FTT and 3 during rest) were acquired from all subjects over the first half of scanning. Additional 3 FTT images and 3 rest images were acquired from 27 of the subjects, totaling 12 sequences (Fig. 5*a*). A statistically significant effect of time on T_1_-weighted estimates of GMV was detected for both protocols. A statistically significant decrease in estimates of GMV was detected already after 12 min of FTT training (including 12 min of resting) in the right thalamus and in the right insular cortex, whereas estimated GMV increased in the left lingual gyrus and in the right superior lateral occipital cortex (sLOC) (Table S3; Fig. 5*b*). Additional changes in GMV estimates were observed during the longer protocol (n = 27) with decreases in GMV estimates from pretraining to 16, 20, and 26 min of training, but not when comparing estimated GMV after 16 min to GMV after 20–26 min of training (Fig. 5*c*, *d*; Table S4, Appendix B, Supplementary Information). GMV decreases were located in the insula bilaterally, in the left paracingulate gyrus, in the right caudate and in the cerebellar vermis VI. No statistically significant time × condition interaction was found, indicating that the pattern of GMV changes over time was similar in the rest and FTT conditions.

#### Do FTT-Induced Changes in BOLD Signal Evolve over 4- and 16 min of FTT Training? (RQ_4_)

**Figure 6:**
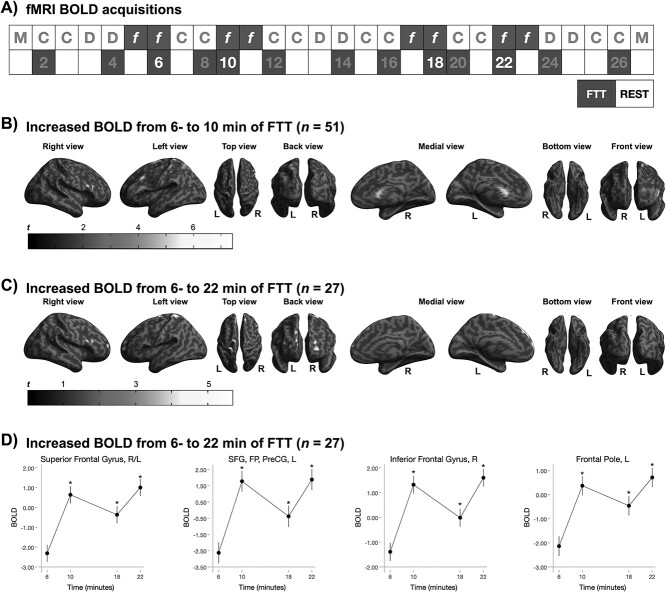
Increased task-related BOLD signal (FTT vs. rest) over of 4- and 16-minute FTT-training. (**A**) Four fMRI sessions (2 FTT, 2 rest) were acquired on all subjects (*n* = 51), for a total of 4-minute FTT-training; additional four sessions (2 FTT, 2 rest) were acquired from 27 subjects, for a total of 16-minute training. **(B)** Whole-brain, voxel-wise BOLD signal during FTT (compared with rest) increased over 4 min of training (*n* = 51, from 6- to 10-min) in frontal and prefrontal areas, in the right superior lateral occipital cortex and right superior temporal gyrus. **(C)** Further, an increase in frontal task-related activity was observed after additional FTT training (*n* = 27, 6 min vs. 22 min). Significant clusters are overlaid to the surface rendering provided by SPM 12. **(D)** Task-related activity (compared with rest) increased from 6-to-10 min and remained higher over the following acquisitions. Error bars represent the standard error. Clusters with *P* < 0.001 (uncorrected) that further survived FWE-correction for multiple testing at cluster level (*P* < 0.05) are reported. An alternative figure in color is available at https://github.com/neuronsson  **Abbreviations**: BOLD, bold-oxygen level-dependent; FTT, finger-tapping task; FWE, family-wise error; L, left; PreCG, precentral gyrus; R, right; SFG, superior frontal gyrus; SPM, statistical parametric mapping.

A statistically significant effect of session was detected over 4- and 22-minute FTT-training. There was large overlap between regions detected at the short and long protocols, though the effect was stronger and more widespread when including all subjects (*n* = 51, 6–10 min). Brain activity remained stably higher after 10 min, such that it was still higher after 18-minute training compared with 6 min, while no differences could be observed from 10 min onward (Fig. 6; Table S5, Appendix B, Supplementary Information).

### Associations between BOLD Signal and T_1_-Based Estimation of GMV (RQ_5_)

**Figure 7:**
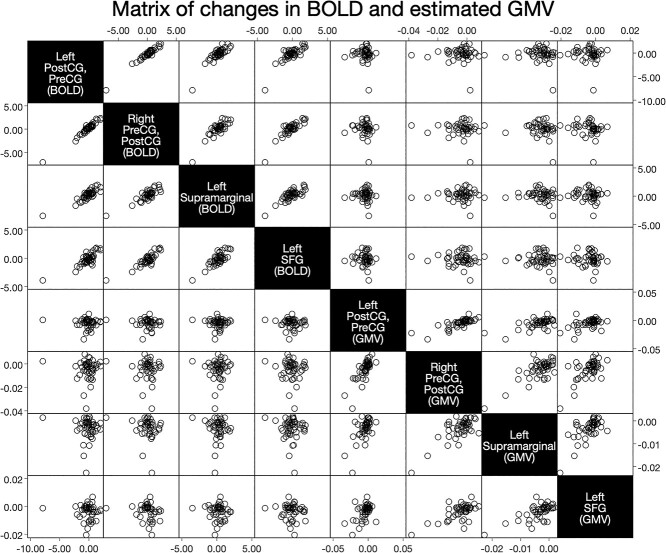
Associations between BOLD signal and estimated GMV. The matrix exemplifies the lack of detectable association between estimated GMV (FTT vs. rest) and BOLD signal. Data in the matrix was generated by extracting GMV and BOLD values from the clusters where a statistically significant effect of condition (FTT vs. rest) was observed on GMV. The matrix has representative purposes only, as the correlation analysis was run voxel-wise whole-brain, testing for voxel-to-voxel correlations between BOLD and GMV. **Abbreviations**: BOLD, blood-oxygen level-dependent; FTT, finger-tapping task; GMV, gray matter volume; PreCG, precentral gyrus; PostCG, postcentral gyrus; SFG, superior frontal gyrus.

No statistically significant associations between task-related changes in GMV estimates (FTT vs. rest) and task-related changes in BOLD signal (FTT vs. rest) were detected at voxel-level (*p* TFCE-corrected ≥0.888). Moreover, no statistically significant associations between time-related (pre- to post-test) T_1_-based GMV estimates and BOLD signal changes were found (*p* TFCE-corrected ≥0.208), suggesting that the observed GMV difference between FTT and rest was not related to any BOLD signal changes (see also Fig. 7).

### Associations between Behavioral Performance and Changes in Estimated GMV or BOLD Signal (RQ_6_)

**Figure 8:**
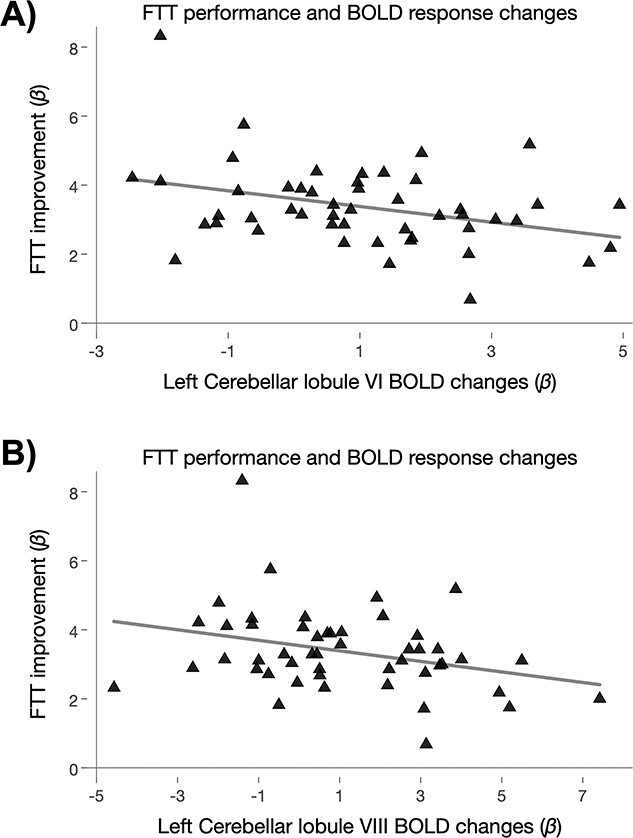
Associations between FTT performance improvements and BOLD signal changes. (*a*) Depicts changes in the left cerebellar lobule VI BOLD signal (during the first half of the scanning protocol, *n* = 51), being associated with FTT improvements during the first half of the protocol (7 FTT sessions in total). Similarly, (*b*) depicts the left cerebellar lobule VIII BOLD signal changes being associated with behavioral performance. Both regions showed that a greater improvement in FTT was associated with a reduction in BOLD signal. **Abbreviations**: BOLD, blood-oxygen level-dependent; FTT, finger-tapping task.

In both modalities (BOLD and T_1_-w estimated GMV), among regions that displayed an effect of either FTT performance increases (changes across time) or condition (difference between FTT and rest), the left cerebellar BOLD signal change was associated with improvements in FTT performance. As displayed in Figure 8, individuals with the largest improvements in motor-skill learning showed a reduction in the left cerebellum BOLD response (from pretraining to 14 min of FTT; Fig. 8*a*, lobule VI, *β* = −0.34, *P* (permuted) = 0.015; Fig. 8*b*, lobule VIII, *β* = −0.31, *P* (permuted) = 0.036). No statistically significant correlations were found between behavioral performance and GMV.

## Discussion

We investigated rapid effects of motor-skill learning on T_1_-weighted estimates of GMV and BOLD-fMRI estimates of neural activity in subjects undergoing 12–26 min of a complex FTT using their left nondominant hand. Motor-related brain regions showed both task-related (FTT vs. rest) and time-dependent changes in brain measures (see also Box 1 for a summary of key findings). Interestingly, time-dependent effects were located in different regions relative to the task-related effects on estimated GMV, and no overlap was observed between estimated GMV and BOLD signals.

Primary motor and sensorimotor cortices (i.e., the right precentral and postcentral gyri) showed statistically significant task-related changes in both estimates of GMV and neural activity. Neural activity was higher in these areas during FTT (relative to resting), whereas GMV was larger in motor-related cortices while participants were resting. Whether fluctuations in the BOLD signal may be responsible of these apparent changes in tissue volume is so far unclear. Despite being located in similar/neighboring cortices, T_1_-weighted estimates of GMV and neural activity did not overlap nor correlate at voxel-level. Moreover, as expected, neural activity was higher in the FTT condition compared with rest, whereas the opposite pattern was observed for T_1_-weighted estimates of GMV, which is not consistent with the notion that higher BOLD signal translates into apparent larger tissue volume measure with T_1_-weighted imaging (Ge et al. 2017). However, different amounts of training were undertaken in-between T_1_-weighted images and BOLD-fMRI sequences, with variable intervals between the acquisitions, limiting our possibility to make direct comparison. Therefore, we cannot rule out with absolute certainty that cerebral BOLD signal changes may have confounded our GMV estimates.

Nevertheless, the possibility that rapid (minutes-wise) GMV changes may not solely derive from BOLD signal alteration needs further exploration. BOLD signal may be the major contributor to apparent changes in estimated GMV while executing passive tasks, when no specific attentional or learning process is required (Månsson et al. 2020) and thus no need for structural reorganization is elicited. Performing active tasks that tap into complex processes such as fine motor learning, if effective in inducing structural plasticity as theorized, might be associated with additional BOLD-independent, true fluctuations in estimated GMV, limiting the room for detecting potential associations between T_1_-based estimates of GMV and BOLD signal changes. This hypothesis was already proposed for cortical thickness, as cortical thickness increases seem not to be driven solely by changes in cerebral blood flow (Taubert et al. 2016). During fast motor learning, a phenomenon called spike-timing-dependent plasticity occurs (Yger et al. 2015). This consists in a precise pace in spiking, with synapses firing following specific, nonrandom time pattern in response to the stimulus. In this context, more efficient synapses are characterized by a more regular, predictive spiking pattern in response to the relevant stimulus (Yger et al. 2015). Pruning of synapses with high-error spikes can reduce the noise in the network and improve performance, speeding up the learning process (Spiess et al. 2016). The timescale of this process is compatible with that of our protocol (Yger et al. 2015; Spiess et al. 2016), indicating that this denoising process may occur during the rest interval between FTT execution acquisitions, when the network is not in use, leading to a reduction in GMV. However, if this hypothesis was true, we would have expected the GMV estimates difference between conditions to also change over time with progressive skill-acquisition and circuit-refinement, which was not the case in our samples. The sensitivity of the inversion preparation sequences (both MPRAGE and wave-CAIPI) to the VASO (Vascular-Space-Occupancy) effect (Lu and van Zijl 2012) might also have partly confounded our results. The VASO is an inversion-nulling technique for detecting changes in cerebral blood volume (CBV) rather than blood oxygenation in neural activation. It exploits the T1 difference between blood and tissue to null the intravascular blood signal and produce an image of extravascular tissue water. Under optimal experimental settings, VASO effect can typically result in less than 1.5% signal reduction at 3 T in brain regions partially overlapping or adjacent to BOLD activations. The wave-CAIPI protocol used in the study was optimized for the VBM measurement (maximum GM/WM contrast) not for VASO effect. As the used TI = 900 ms is larger than the blood signal nulling timepoint (~690 ms), blood will contribute positively to the wave-CAIPI images. However, T1 for blood is slightly longer than T1 for GM, the vascular blood water signal will be relatively smaller than that for the extravascular tissue water. The CBV increase associated with FTT will lead to a slightly reduced GMV estimate. However, this cannot fully account for the observed GMV reduction. The detected GMV change in the study does not overlap with the BOLD signal variations and the expected VASO effect in the current experimental setting should be much less than what was observed in an optimal VASO protocol. Larger movement during task execution might also lead to apparent reductions in estimated GMV during FTT compared with rest (Reuter et al. 2015); however, we found no statistically significant effects on condition (FTT vs. rest; *P* = 0.148), time (*P* = 0.138), or a condition × time interaction (*P* = 0.627) related to quality rating of the T1-weighted CAIPI images. Moreover, no effects of condition (FTT vs. rest; *P* = 0.227), time (*P* = 0.427), or condition × time interaction (*P* = 0.736) were detected on frame-wise displacement during the fMRI acquisition (not reported). As presented in the Supplementary Information, the test–retest reliability of the wave-CAIPI acquisitions was also excellent, with ICC relative to intracranial brain volume of 0.92 in wave 1 and 0.94 in wave 2 (Koo and Li 2016). Future studies using fast-learning protocols are warranted to further explore the anatomical–functional correlates of rapid task-induced effects on estimates of GM measures.

Time-dependent effects were observed in the right sLOC (part of the extrastriate visual path), in the insula, and in subcortical motor-related areas, namely the thalamus and caudate nucleus. The LOC is essential for allocentric visual coding, that is, for the execution of movements based on allocentric visual information (e.g., drawing or copying) as opposed to target-directed movements (e.g., pointing or reaching) (Thaler and Goodale 2011). The extrastriate LOC responds to goal-directed movements of the observer’s body parts (Astafiev et al. 2004), and activity in this region is triggered by the imitation of previously observed movements, rather than by pure, passive observation (Jackson et al. 2006). The sLOC exhibited structural and functional changes. Although no spatial overlap between structural and functional changes was observed, the direction of such changes was nonetheless consistent for both T_1_-weighted and BOLD-fMRI signal. A progressive increase in GMV estimates was observed after 12-minute training independent of condition (FTT or rest), paralleled by increasing task-related neural activation over time (i.e., the activity during FTT compared with rest increased over time). This pattern of changes suggests that GMV measurements in the sLOC may be at least partly influenced by underlying BOLD signal fluctuations.

On the other hand, the posterior insula, thalamus and caudate only exhibited reduced estimates of GMV, with no parallel changes in BOLD signal. The right insula and thalamus had smaller GMV estimates already after 12-minute training, whereas GMV estimates in the right caudate were progressively reduced during the 26-minute training. The insula, thalamus andcaudate are strongly involved in motor control and motor learning (Sommer 2003; Haber 2016; Varjacic et al. 2018; Driscoll et al. 2020). Their role in motor-skill learning, coupled to the lack of detectable BOLD signal fluctuations paralleling the reduction in GMV estimates, indicates that structural plasticity may be at play in these areas. The insula functions as a hub for several higher cognitive function, including the modulation of attentional and salience processing (Uddin et al. 2017), and is necessary for goal-directed behaviors (Varjacic et al. 2018). The posterior insula in particular is widely connected with sensorimotor regions (Cauda et al. 2012; Evrard and Craig 2015), the supplementary motor area (Evrard and Craig 2015) and the thalamus (Ghaziri et al. 2018), which is a central hub for motor control (Sommer 2003). The thalamus is intercalated on a thalamo-cortico-striatal circuit (Sommer 2003; Haber 2016) that is crucial for the acquisition and execution of learned motor sequences (Haber 2016). The right caudate receives inputs from the striatum and is part of the core control system for skeletal movements with the basal ganglia (Driscoll et al. 2020). The caudate is involved in planning the execution of movement, but is also essential for skill learning (Poldrack 2002; Choi et al. 2020), playing a crucial role in connecting visual stimuli with motor responses and in feedback-aided learning (Jueptner et al. 1997; Poldrack 2002; Driscoll et al. 2020). Neural activity in the caudate, as well as its functional connectivity with sensorimotor cortices, predicts individual learning performance in a simple motor task (Choi et al. 2020). Further, structural plasticity has been reported to occur in the caudate of mice brains after prolonged motor training (Badea et al. 2019).

**Box 1. Key findings**

- Task-related changes in T_1_-based estimates of GMV in motor and somatosensory cortices can be detected during FTT execution, and these apparent tissue changes are somewhat different from other time-related modifications occurring over repeated 12–26-minute FTT-training.
- Larger GMV while resting (as compared with FTT) indicates that engaging in a task prior to anatomical imaging may induce morphological changes.
- BOLD signal and estimated GMV changes are not overlapping and not correlated. Thus, nearly simultaneous changes in BOLD signal cannot fully explain rapid changes in GMV estimates based on T_1_-weighted signal.

We performed whole-brain voxel-wise analyses (corrected for multiple testing) and report motor-related regions that show both statistically significant effects of the task (FTT vs. rest) and time-related effects, supporting the validity of our protocol and findings. However, rapid effects on estimated GMV in these regions were not associated with the number of accurately completed sequences in the FTT. Indeed, we found only 2 scarce correlations between FTT performance improvement and increased neural activations in the left cerebellum. Of note, the lobules VI and VIII of the cerebellum contribute to sensorimotor control (Stoodley and Schmahmann 2010) and have been involved in ipsilateral FTT (Batson et al. 2015) and hand-eye coordination (Batson et al. 2015), respectively. The lack of other detectable correlations may be partly due to interindividual differences in learning strategies and pace. Individual variability in the strategies adopted during motor-skill learning, and the rate of improvement on task performance, can in fact differently lead to bidirectional changes in GMV estimates, such that GMV estimates can either increase or decrease based on the strategy used (Gryga et al. 2012).

Our protocol consisted of multiple repeated acquisitions of T_1_-weighted and BOLD-fMRI volumes, allowing us to detect the time course of changes in measures of brain structural and functional activity. However, some limitations have to be noted. First of all, our study did not include a control group assigned to a nonmotor control condition. The lack of this comparison might pose the question as to whether the observed time-related changes are directly due to the motor task or to other confounding factors occurring over time, such as experience in the scanner. Nonetheless, our main RQ concerned the difference in estimated GMV while individuals executed a FTT and while resting. This question could be adequately addressed by our protocol, employing a randomized within-subject, between-condition design. The localization of the GMV (and BOLD signal) changes, mainly limited to the primary motor and somatosensory cortices, further support the notion of specific motor-related effects. Indeed, participants with the double amount of FTT practice (60 min, study wave 1), showed in general stronger effects in similar brain regions as individuals assigned to the shorter protocol (30 min, study wave 2). We used nearly simultaneous multimodal imaging, taking a step forward in the investigation of the complex relationship between task-induced changes in T_1_-weighted and fMRI-BOLD signal; however, the structural and functional acquisitions were not simultaneous, and different amounts of training were undertaken in-between wave-CAIPI and fMRI sessions, with variable intervals between the acquisitions. Moreover, we did not acquire pretest measurements of neural activity. Further studies, using combined structural MRI and simultaneous measurements of blood flow fluctuations and neural activity (as measured by functional near-infrared spectroscopy, for example), may shed further light on the association between T_1_-based estimates of GMV and BOLD signal fluctuations. The use of wave-CAIPI sequences for T_1_-weighted structural MRI acquisitions is not common; however, it allowed us to acquire repeated measurements of brain morphology as fast as 2-minute apart. A direct comparison of the wave-CAIPI with the most common MPRAGE acquisition showed high concordance between the 2 methods, with a significant positive correlation in 99.5% of the voxels at pretraining (98.9% voxels with *r* ≥ 0.872), and in 93.0% of the voxels at post-training (90.7% voxels with *r* ≥ 0.832) (see also Appendix C, Supplementary Material). It should also be mentioned that the subjects were not randomized to the shorter or longer training protocols, but were rather recruited in waves; however, no statistically significant group differences in FTT performance were detected across timepoints (*P* > 0.143), and performance improvement over time followed the same pattern in both groups.

With rapid parallel imaging techniques, we acquired multiple repeated volumes of T_1_-weighted estimated GMV and BOLD-fMRI measurements during the execution of 2 min of FTT alternated similar acquisitions of rest over 30- and 60-minute scanning time. Several motor-related regions showed task-related and time-dependent changes in brain measures. Rapid task-related changes in T_1_-based estimates of GMV and neural activity were detected in the primary motor and somatosensory cortices at voxel-wise, whole-brain analyses. Estimates of GMV were larger during rest, as compared with FTT, indicating that engaging in a task during anatomical imaging may induce apparent morphological changes. Neural activity was, as expected, higher during FTT than rest, in contiguous but not overlapping clusters of the same regions. Task-related estimates of GMV and BOLD-fMRI were not correlated in a voxel-wise way. Time-dependent effects (12–26 min) during motor-skill learning were mostly located in different regions than those involved in task-related effects (FTT vs. rest), namely areas linked to motor learning rather than motor execution. Estimated GMV increased in the insula and thalamus after 12 min of training, and in the caudate over 26 min of training. These changes were, however, not overlapping and not correlated with BOLD-estimated neural activation changes.

We conclude that nearly simultaneous changes in blood flow could not fully explain the apparent and rapid changes in GMV estimates based on T_1_-weighted imaging. Further, task-related (FTT vs. rest) changes did not overlap with time-related plasticity, hinting at some structural plasticity mechanisms playing a role in determining such changes. Although we cannot make conclusive statements concerning the nature of the observed GMV changes, we can nonetheless argue that common estimates of GMV with T_1_-weighted MRI are sensitive to in-scanner behavior, and may thus pose a serious threat to reproducibility across studies, acting as a confounder. However, this also suggests that the combined use of task-related structural and functional imaging could be a new frontier to advance our knowledge on rapid brain plasticity.

## Authors’ Contribution

KM, HF, ML, and TL planned and designed the study. KM, AM, LT, BJ, AJ, TL, and SP collected the data. GO and KM analyzed the data. GO, KM, HF, ML, and TL interpreted the data. GO and KM drafted the manuscript. All authors revised the manuscript and approved the submitted version of the work.

## Funding

The Swedish Research Council (2018-01047).

## Notes

The datasets generated during and/or analyzed during the current study are not publicly available due to general data protection regulation (GDPR) and ethics restrictions, but are available from the corresponding author on reasonable request. *Conflict of Interest:* The authors declare no conflicts of interest. There have been no involvements that might raise the question of bias in the work reported or in the conclusions, implications, or opinions stated.

## Supplementary Material

Gaia_etal_Cerebral_Cortex_Supplementary_information_bhab488
