# Supplementary material for: Estimated gray matter volume rapidly changes after a short motor task
Source: Cereb Cortex. 2022 Feb 8;32(19):4356–69. doi: 10.1093/cercor/bhab488 (PMC9528898; doi:10.1093/cercor/bhab488)
Supplement: Gaia_etal_Cerebral_Cortex_Supplementary_information_bhab488 [file gaia_etal_cerebral_cortex_supplementary_information_bhab488.docx]

**Supplementary Information**

**Estimated gray matter volume rapidly changes after a short motor task**

*Gaia Olivo^1,2^, Martin Lövdén^1,2^, Amirhossein Manzouri^3,4^, Laura Terlau^5,6^, Bo Jenner^4^, Arian Jafari^4^, Sven Petersson^7,8^, Tie-Qiang Li^7,8^, Håkan Fischer^3,9^, Kristoffer N . T. Månsson^4,10,3*^*

*^1^* *Department of Psychology, University of Gothenburg, Gothenburg, Sweden*

*^2^ Aging Research Center (ARC), Department of Neurobiology, Care Sciences and Society, Karolinska Institutet, Stockholm, Sweden*

*^3^ Department of Psychology, Stockholm University, Stockholm, Sweden*

*^4^ Centre for Psychiatry Research, Department of Clinical Neuroscience, Karolinska Institutet, Stockholm, Sweden*

*^5^ Center for Lifespan Psychology, Max Planck Institute for Human Development, Berlin, Germany*

*^6^ Max Planck UCL Centre for Computational Psychiatry and Ageing Research, Berlin/London*

*^7^ Department of Medical Radiation and Nuclear Medicine, C2-76, Karolinska University Hospital, Huddinge S-141 86 Stockholm, Sweden*

*^8^ Department of Clinical Science, Intervention and Technology, Karolinska Institutet, Stockholm, Sweden*

*^9^ Stockholm University Brain Imaging Centre, Stockholm, Sweden*

*^10^ Department of Psychological and Brain Sciences, Dartmouth College, Hanover, NH, USA*

* Address for correspondence: Dr. Kristoffer N. T. Månsson, Centre for Psychiatry Research, Department of Clinical Neuroscience, Karolinska Institutet; Norra Stationsgatan 69, SE-113 64 Stockholm, Sweden; Tel: +46(0)705803267; E-mail: kristoffer.mansson@ki.se; kristoffer.nt.mansson@dartmouth.edu

**Appendix A. Demographic information, self-rated health ratings and handedness of the participants.**

| **Table S1.** Demographics, self-rated health and handedness. | | |
| --- | --- | --- |
| Age: average, SD | 26.1 | 4.0 |
| Females, *n*, % | 27 | 51.9 |
|  |  |  |
| **Education, highest degree** | ***n*** | ***%*** |
| University | 12 | 23.1 |
| High school | 37 | 71.2 |
| Vocational/Other | 3 | 5.8 |
|  |  |  |
| **SRH-7** | ***n*** | ***%*** |
| Excellent | 18 | 34.6 |
| Good | 26 | 50.0 |
| Mostly good | 8 | 15.4 |
|  |  |  |
| **EHI (Responding “commonly” or “always” using the right hand)** | ***n*** | ***%*** |
| Writing | 52 | 100.0 |
| Drawing | 52 | 100.0 |
| Throwing | 52 | 100.0 |
| Scissors | 52 | 100.0 |
| Tooth brushing | 50 | 96.2 |
| Knife (without fork) | 49 | 94.2 |
| Spoon | 51 | 98.1 |
| Comb | 48 | 92.3 |
| Striking a match | 52 | 100.0 |
| Opening a box (lid) | 44 | 84.6 |
|  |  |  |
| **Abbreviations:** EHI, Edinburgh Handedness Inventory | | |

**Appendix B. Effect of condition on estimated GMV and BOLD signal.**

**
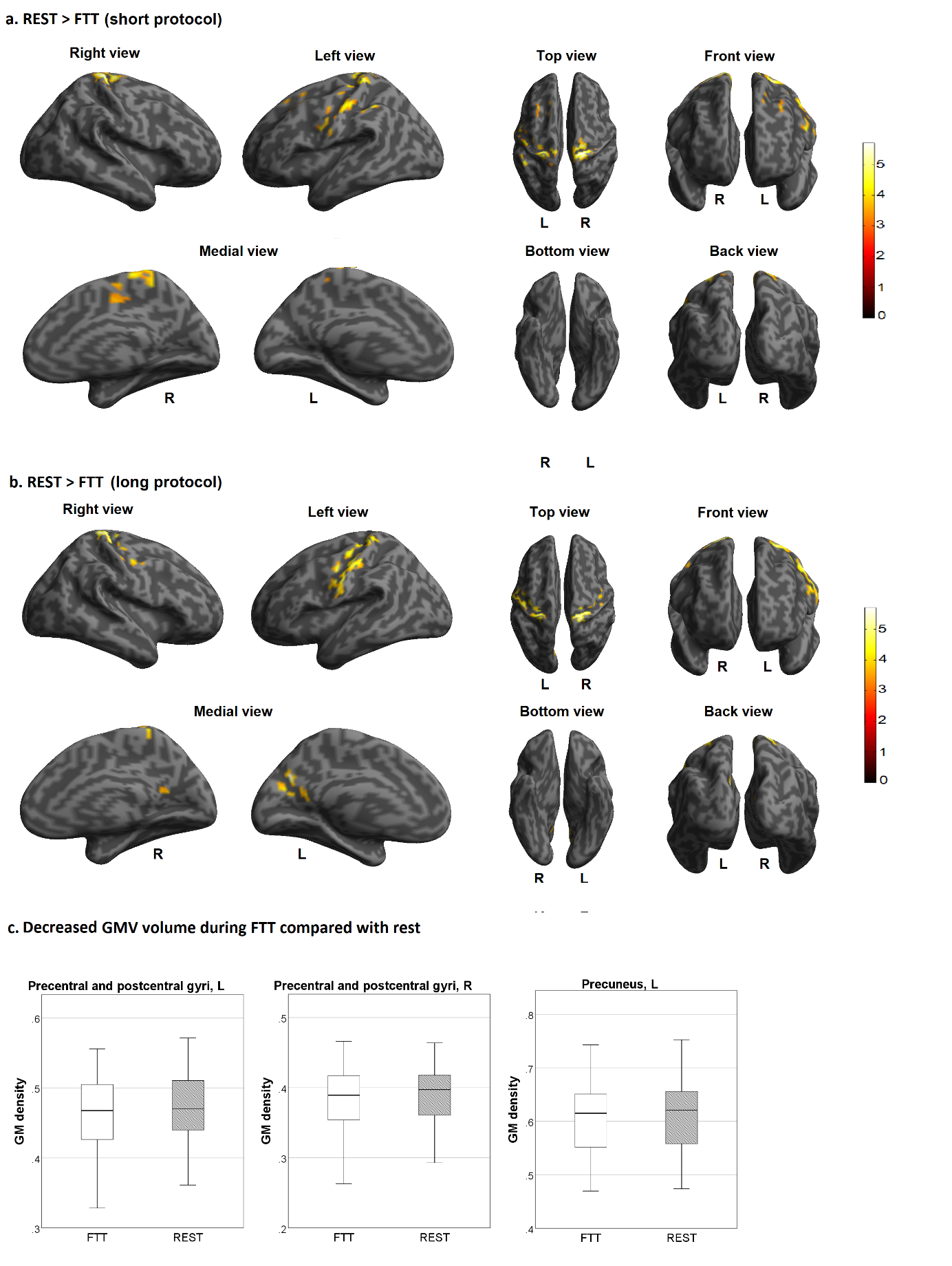
**

**
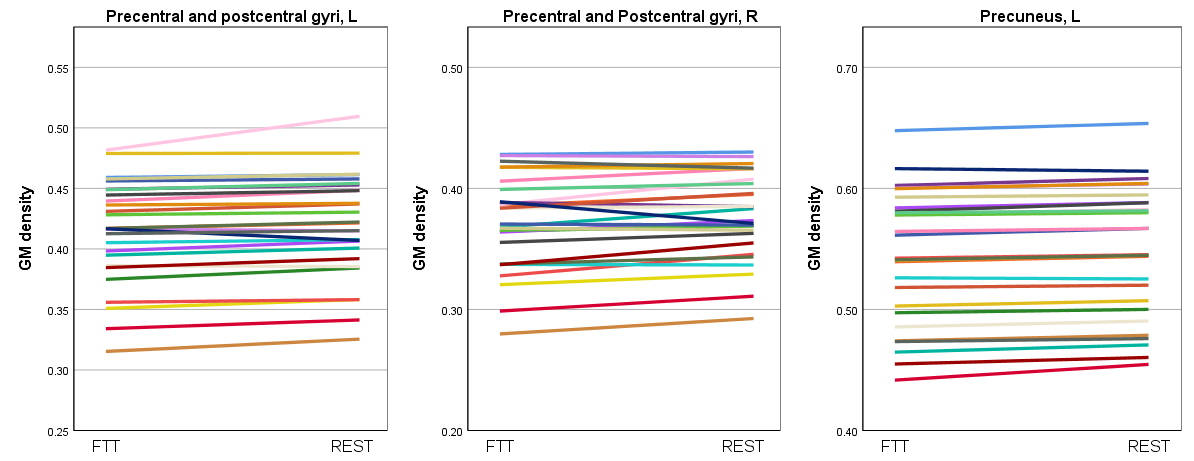
**

**Figure S1. Effects of 2-minutes task execution on estimates of GMV.** The figure shows clusters where statistically significant differences in estimated GMV between the 2-minutes FTT blocks and the 2-minutes resting blocks were detected over (a) the 30-minutes (n=51) and (b) 60-minutes (n=27) training protocols. Estimates of GMV in the depicted clusters were increased during rest compared with the execution of the FTT (c). Clusters with *p* < .001 at the uncorrected level that further survived FWE-correction for multiple testing at cluster level at *p* < .05 are reported. Significant clusters are overlaid to the surface rendering provided by SPM 12. Error bars (c) represent standard error.

**Table S2. Effect of 2-minutes FTT execution on grey matter volume.**

| p FWE | Ce | F | MNI coordinates (maxima) | Direction | Structure |
| --- | --- | --- | --- | --- | --- |
| *Main effect of condition (2-minutes; FTT vs rest), shorter protocol (n=51)* | | | | | |
| <.001 | 2220 | 36.83 | 19, -27, 70 | Rest > FTT | R PreCG, PostCG |
| <.001 | 1704 | 33.04 | -55, -42, 48 | Rest > FTT | L Supramarginal gyrus |
| <.001 | 1201 | 26.18 | -24, -30, 66 | Rest > FTT | L PostCG, PreCG |
| .004 | 745 | 23.15 | -24, 22, 51 | Rest > FTT | L SFG |
|  | | | | | |
| *Main effect of condition (2-minutes; FTT vs rest), longer protocol (n=27)* | | | | | |
| <.001 | 2664 | 26.66 | -41, -18, 49 | Rest > FTT | L PreCG, PostCG |
| <.001 | 1383 | 29.73 | 20, -31.5, 70 | Rest > FTT | R PostCG, PreCG |
| <.001 | 1216 | 30.79 | -9, -69, 29 | Rest > FTT | L Precuneus |

**Abbreviations**: Ce, cluster extent; F, F-score; FWE, family-wise error; L, left; PreCG, precentral gyrus; PostCG, postcentral gyrus; R, right

**
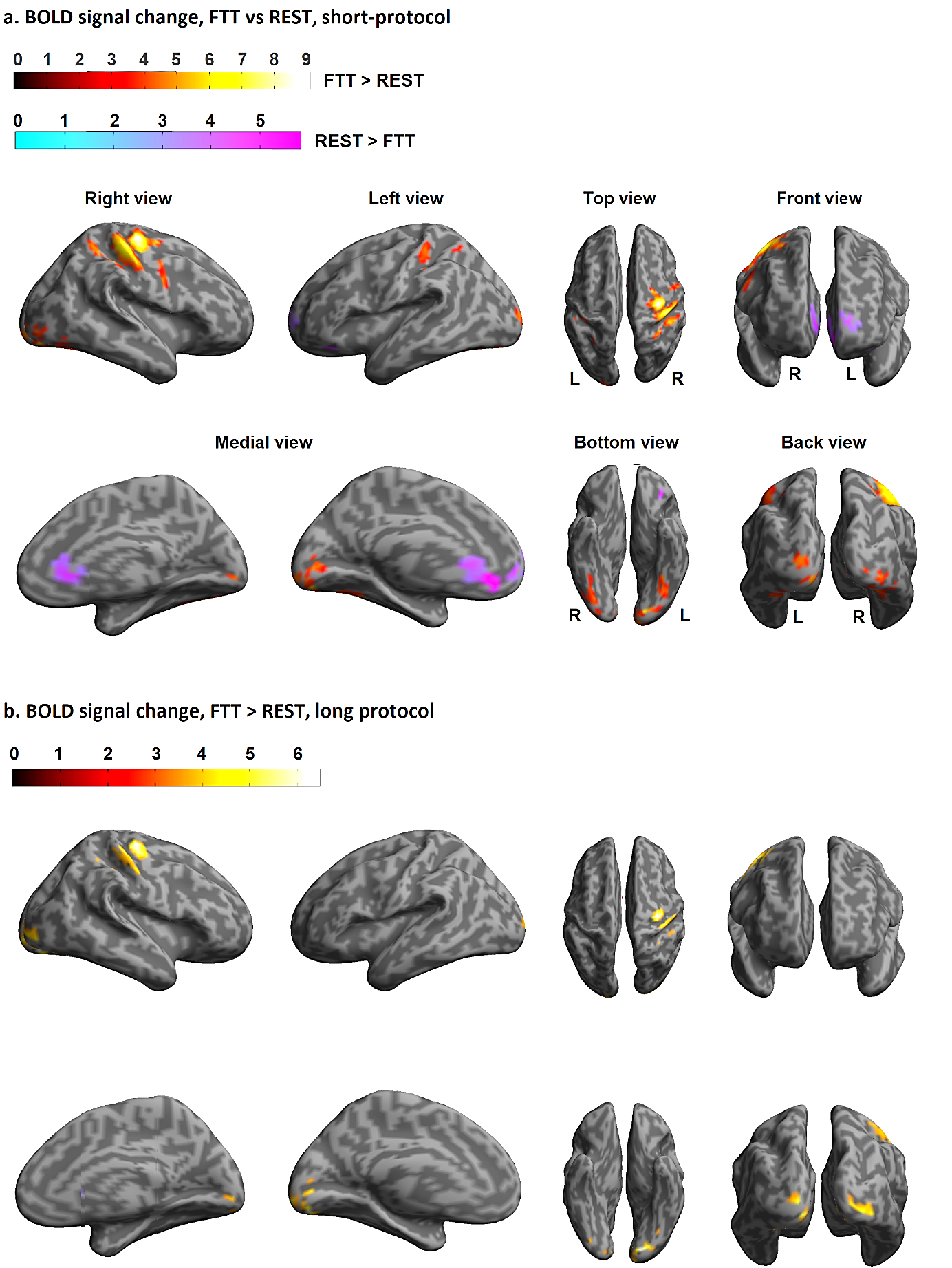
**

**Figure S2. Effects of the FTT task on BOLD signal.** The figure shows clusters where the FTT task elicited significantly higher functional activity compared with rest over (a) the short (n=51) and (b) long (n=27) training protocols. The FTT-REST difference was bidirectional at the short protocol (a), while only the FTT > REST contrast yielded statistically significant results at the long protocol (b). Clusters with *p* < .001 at the uncorrected level that further survived FWE-correction for multiple testing at cluster level at *p* < .05 are reported. Significant clusters are overlaid to the surface rendering provided by SPM 12.

**Table S3. Brain activity difference during FTT execution compared with rest (task effect).**

| p FWE | Ce | | F | | MNI Coordinates | | Direction | | Structure |
| --- | --- | --- | --- | --- | --- | --- | --- | --- | --- |
| *Main effect of condition, short training protocol* | | | | | | | | | |
| <.001 | | 4751 | | 63.21 | | -24, -54, -22 | FTT > rest | L Cerebellar lobule VI | |
| <.001 | | 2478 | | 91.69 | | 38, -20, 62 | FTT > rest | R PreCG, PostCG | |
| <.001 | | 758 | | 46.32 | | -26, -56, -48 | FTT > rest | L lobule VIII | |
| .037 | | 246 | | 35.64 | | -56, 0, 42 | FTT > rest | L PreCG | |
| .008 | | 361 | | 22.38 | | -46, -34, 52 | FTT > rest | L PostCG, SPL | |
| <.001 | | 1567 | | 46.36 | | -34, 34, -8 | Rest > FTT | L OFC, L Frontal pole | |
|  | |  | |  | |  |  |  | |
| *Main effect of condition, long training protocol* | | | | | | | | | |
| <.001 | | 1631 | | 59.29 | | -14, -86, -14 | FTT > rest | L Fusiform, lingual, OP, lobule VI | |
| <.001 | | 680 | | 45.88 | | 38, -20, 62 | FTT > rest | R PreCG, PostCG, supramarginal gyrus | |
| .005 | | 403 | | 27.32 | | -6, -68, -44 | FTT > rest | R OP, iLOC | |
| .006 | | 385 | | 31.40 | | 28, -96, -2 | FTT > rest | L lobule VIII, lobule IX | |

**Abbreviations**: Ce, cluster extent; F, F-score; iLOC, inferior lateral occipital cortex; L, left; OFC, orbitofrontal cortex; OP, occipital pole; PreCG, precentral gyrus; PostCG, postcentral gyrus; SPL, superior parietal lobule; R, right

**Table S4. Estimated GMV changes over 12 and 26-minutes training (time effect).**

| p FWE | Ce | F | MNI coordinates (maxima) | Direction | Structure |
| --- | --- | --- | --- | --- | --- |
| *Main effect of run, 12-minutes training (n=51)* | | | | | |
| .026 | 488 | 26.31 | 8, -13, 0 | 0’ > 12’ | R Thalamus |
| .047 | 419 | 30.05 | 43, 15, -11 | 0’ > 12’ | R Insula, Temporal Pole |
| .007 | 666 | 23.37 | 29, -72, 43 | 12’ > 0’ | L sLOC, Angular Gyrus |
| .033 | 461 | 25.20 | -11, -66, -11 | 12’ > 0’ | L Lingual gyrus, Lobule VI |
|  |  |  |  |  |  |
| *Main effect of run, 26-minutes training (n=27)* | | | | | |
| <.001 | 2313 | 53.26 | 45, 15, -7 | 0’ > 16’-26’ | R Insula, Heschl’s Gyrus |
| <.001 | 1408 | 37.13 | -43, 12, -9 | 0’ > 16’-26’ | L Insula, Frontal Operculum |
| <.001 | 1058 | 28.01 | -2, 48, -9 | 0’ > 16’-26’ | L Paracingulate, MedFC |
| .010 | 579 | 73.43 | 9, 21, 4 | 0’ > 16’-26’ | R Caudate |
| <.001 | 2268 | 31.46 | -3, -73, -22 | 26’ > 0’ | Vermis VI, Crus I |

**Abbreviations**: Ce, cluster extent; F, F-score; FWE, family-wise error; L, left; MedFC, medial frontal cortex; R, right; SFG, superior frontal gyrus; sLOC, superior lateral occipital cortex

**Table S5. Effects of 4- and 16-minutes FTT training on brain activity.**

| p FWE | Ce | F | MNI coordinates (maxima) | Direction | Structure |
| --- | --- | --- | --- | --- | --- |
| *Main effect of run, 4-minutes training (n=51)* | | | | | |
| <.001 | 41233 | 84.88 | -54, 16, 34 | 10’ > 6’ | L/R MFG, L/R IFG, R/L Caudate |
| .003 | 505 | 27.57 | 34, -86, 18 | 10’ > 6’ | R sLOC, R Occipital Pole |
| .004 | 474 | 33.15 | 66, -22, 6 | 10’ > 6’ | R STG, Angular gyrus |
|  |  |  |  |  |  |
| *Main effect of run, 16-minutes training (n=27)* | | | | | |
| <.001 | 2093 | 28.33 | 0, 34, 54 | 10’-22’ > 6’ | R/L SFG |
| <.001 | 841 | 19.09 | -8, -8, 76 | 10’-22’ > 6’ | L SFG, PreCG, PostCG |
| .025 | 358 | 26.92 | 58, 20, 26 | 10’-22’ > 6’ | R IFG, MFG, PreCG |
| .049 | 293 | 17.90 | -20, 54, 4 | 10’-22’ > 6’ | L FP, SFG, PreCG, PostCG |

**Abbreviations**: Ce, cluster extent; F, F-score; FP, frontal pole; FWE, family-wise error; IFG, inferior frontal gyrus; L, left; MFG, middle frontal gyrus; PreCG, precentral gyrus; PostCG, postcentral gyrus; R, right; sLOC, superior lateral occipital cortex; SFG, superior frontal gyrus; STG, superior temporal gyrus

**Appendix C. Validation of the wave-CAIPI sequence**

*Voxel-to-voxel correlations between wave-CAIPI and MPRAGE images*

Prior to investigating our primary research questions (RQ), we explored the consistency between whole-brain, voxel-wise estimates of GMV measured by the gold-standard MPRAGE and by the wave-CAIPI sequences, respectively. The standard MPRAGE was only acquired during rest; therefore, for comparison purposes, the first (run 1) and last (run 6) wave-CAIPI acquisitions in the resting condition were used for this analysis. Voxel-to-voxel correlations between the two modalities were tested in FSL (FMRIB Software Library) (Jenkinson et al. 2012). The “randomise” command implemented in FSL (Winkler et al. 2014) allows for the inclusion of voxel-specific covariates in the analysis via the “--vxl" option. The pre-test MPRAGE was tested for voxel-to-voxel correlations with the first wave-CAIPI acquired in the resting condition (run-1; second scan) in the whole sample. The post-test MPRAGE was tested for voxel-to-voxel correlations with the last wave-CAIPI acquired during rest (run-6; second-last scan for the 60-minutes protocol; last scan for the 30-minutes protocol). FSL provides a non-parametric permutation-based statistics (Smith et al. 2006). The number of permutations was set at 1,000. The threshold for significance was set at *p* < .05, corrected for multiple comparisons at cluster level with a threshold-free cluster enhancement (TFCE) approach (Smith and Nichols 2009).

*Test-retest reliability of wave-CAIPI acquisitions*

Voxel-wise, whole-brain intraclass correlation coefficient (ICC) (Koo and Li 2016) were calculated to measure the test-retest reliability of the wave-CAIPI acquisitions in the FTT and rest conditions respectively. ICC maps were calculated separately in wave 1 and wave 2. The DPABI (Data Processing & Analysis for Brain Imaging) toolbox (<http://rfmri.org/dpabi>) was used for the analysis. An ANOVA model was used. ICC values less than 0.5 are indicative of poor reliability, values between 0.5 and 0.75 indicate moderate reliability, values between 0.75 and 0.9 indicate good reliability, and values greater than 0.90 indicate excellent reliability (Koo and Li 2016). CAT12 also provides quality ratings of the T1w images, accounting for noise contrast ratio, inhomogeneity contrast ratio, and root mean square resolution (http://www.neuro.uni-jena.de/cat12-html/cat_methods_QA.html#Dahnke:2016).

*Results*

*Voxel-to-voxel correlations between wave-CAIPI and MPRAGE images*

Baseline, pre-test GMV estimates obtained with the wave-CAIPI and MPRAGE acquisitions (n=51) were significantly correlated, with p TFCE-corrected < .05 (*r* ≥ .284) for a positive correlation in 99.5% of the voxels (98.9% with p TFCE-corr < .001 and *r* ≥ .872). Most of the voxels were still highly correlated after 60-minutes training (n=27), with 93.0% of the voxels having p TFCE-corrected < .05 (*r* ≥ .666) for a positive correlation (90.7% p TFCE-corr < .001 and *r* ≥ .832). The subjects who underwent 30-minutes training followed by 30-minutes rest also had a statistically significant correlation in 96.4% of the voxels (*r* ≥ .689), with p TFCE-corr < .05 (88.4% with p TFCE-corr < .001 and *r* ≥ .846). In both groups the correlation was weaker for outer cortical GM and in the subcortical structures (e.g. basal ganglia) at the boundaries with white matter. No voxels were negatively associated.


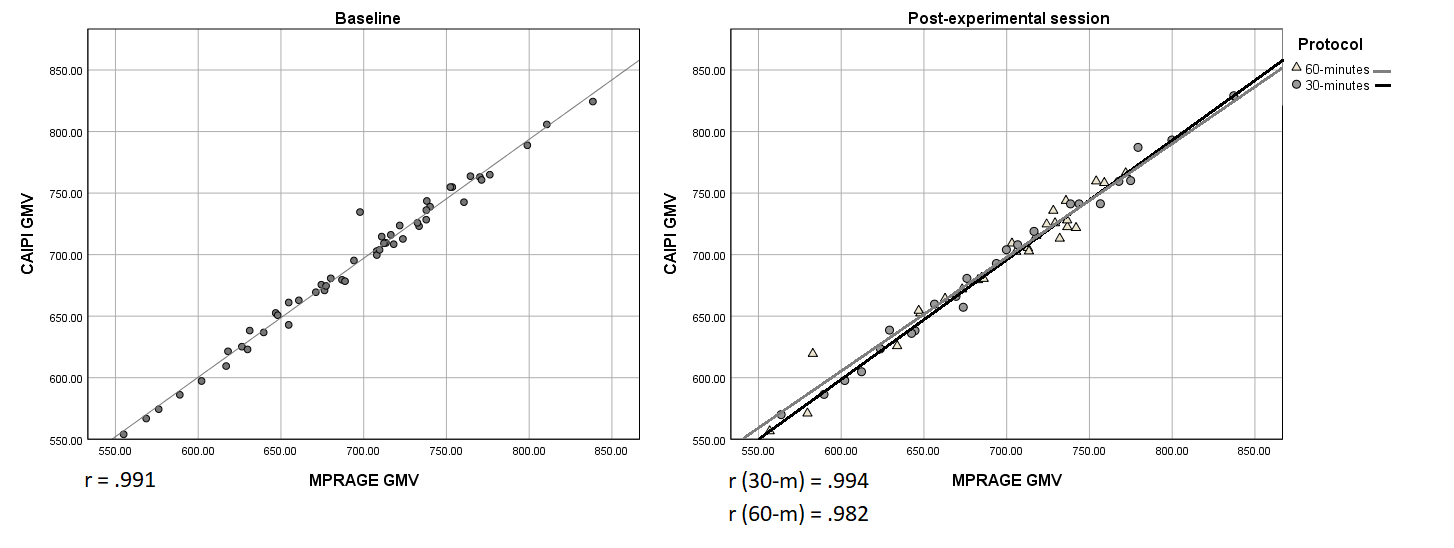


**Figure S3. Correlations between total estimated GMV as measured by wave-CAIPI and MPRAGE sequences**. The scatter plots represent the correlations between total GMV estimated with the wave-CAIPI and MPRAGE acquisitions before (left) and after (right) the experimental session.

*Test-retest reliability of wave-CAIPI acquisitions*

The mean ICC relative to intracranial brain volume during FTT was 0.92 in wave 1 (0-60 minutes) and 0.94 in wave 2 (0-30 minutes), respectively. The mean ICC for images acquired during rest was also 0.92 in wave 1 (0-60 minutes) and 0.94 in wave 2 (0-60 minutes). These values indicate excellent reliability (Koo and Li 2016). No statistically significant effects of condition (FTT versus rest; *p*=.148), time (*p*=.138), or condition × time interaction (*p*=.627) were detected on the T1-weighted CAIPI image quality rating.

**Appendix D. Protocols comparison.**

*Comparison between the short and long protocol*

To investigate differences between the short and long training protocols, a flexible factorial analysis was carried out on the MPRAGE acquisitions. Time (pre-test versus post-test) and protocol were set as within-subjects factors. Main effects of time, protocol, and time*protocol were tested, where protocol and time*protocol were the effects of interest. The primary threshold for significance was set at *p* < .001, uncorrected; voxels surviving the primary threshold were further corrected for multiple testing with a family-wise error (FWE) rate approach at cluster level, with a threshold of *p* < .05.

*Results*

As expected, a statistically significant bidirectional effect of time was detected on several clusters. Estimates of GMV decreased from pre- to post-test in the right insula extending to the right OFC, in the right cerebellar lobule VII, extending to the crus II, in the left insula extending to the parahippocampal gyrus; in the right paracingulate gyrus extending to the frontal pole bilaterally and to the medial frontal gyrus; in the right thalamus; and in the left cerebellar crus I (Table S2). Such effects were, however, independent of the protocol type, as no statistically significant effects of neither the protocol type nor time*protocol interaction were detected.

**Table S6. Decreased estimates of GMV after the experimental session, measured with MPRAGE.**

| Cluster | p FWE | Ce | F | MNI Coordinates | Structure |
| --- | --- | --- | --- | --- | --- |

| 1 | 2424 | < .001 | 7.74 | 38 | 16 | -15 | R insula, orbitofrontal |
| --- | --- | --- | --- | --- | --- | --- | --- |
| 2 | 1775 | .001 | 7.34 | 40 | -68 | -54 | R VIIb, Crus II |
| 3 | 2052 | < .001 | 5.80 | -42 | 12 | -10 | L Insula |
| 4 | 1183 | .008 | 5.57 | 2 | 57 | -3 | R Paracingulate gyrus |
| 5 | 1136 | .010 | 5.09 | 24 | -32 | 2 | R Thalamus |
| 6 | 856 | .029 | 4.67 | -39 | -66 | -34 | L Crus I |

**Abbreviations**: Ce, cluster extent; F, F-score

**Appendix E. Voxel-wise imaging-behavioral correlations**

*Voxel-wise correlations between estimated GMV and BOLD signal changes, and performance improvement.*

Voxel-wise correlations between imaging measures and improvement on behavioral performance were tested with FSL. To test for correlations between performance improvement and the effect of condition on estimated of GMV and BOLD signal, the FTT vs rest contrast was calculated at the first and the last acquisition for each modality (estimated GMV, BOLD). The difference between time-points was computed at each voxel, and the resulting images were used as dependent variable for a regression analysis with performance improvement as covariate of interest. The analysis was masked for the effect of condition.

To test for correlations between performance improvement and the effect of training, estimated GMV and BOLD signal at the first block (averaged conditions for estimated GMV, FTT vs rest for fMRI) and at the last block were calculated, and the voxel-wise difference was computed. The resulting images were used in a regression analysis with performance improvement as covariate of interest. The analysis was masked for the effect of time.

Non-parametric permutation-based statistics for used for all regression analyses. The number of permutations was set at 5,000. The threshold for significance was set at *p* < .05, corrected for multiple comparisons at cluster level with a threshold-free cluster enhancement (TFCE) approach (Smith and Nichols 2009).

*Results*

A small cluster in the left supramarginal cortex (17 voxels) approached significance (p TFCE-corrected = 0.07) for a correlation between estimates of GMV and the slope on performance, such that the larger the difference between rest-GMV and FTT-GMV became over time, the less performance improved over time. BOLD signal also approached significance in the left fusiform gyrus (32 voxels) (p TFCE-corrected = 0.051) when masked for the condition effect, such that the less the task-related activity (compared with REST) changed over time, the more the performance improved; and viceversa, the more task-related activity (compared with rest) increased over time, the less the performance improved, suggesting that subjects with lesser performance gain required more cognitive resources (higher activity) to execute the task.

**References**

Koo, T. K. and M. Y. Li (2016). "A Guideline of Selecting and Reporting Intraclass Correlation Coefficients for Reliability Research." J Chiropr Med **15**(2): 155-163.

Smith, S. M. and T. E. Nichols (2009). "Threshold-free cluster enhancement: addressing problems of smoothing, threshold dependence and localisation in cluster inference." Neuroimage **44**(1): 83-98.

Koo, T. K. and M. Y. Li (2016). "A Guideline of Selecting and Reporting Intraclass Correlation Coefficients for Reliability Research." J Chiropr Med **15**(2): 155-163.

Smith, S. M. and T. E. Nichols (2009). "Threshold-free cluster enhancement: addressing problems of smoothing, threshold dependence and localisation in cluster inference." Neuroimage **44**(1): 83-98.

Koo, T. K. and M. Y. Li (2016). "A Guideline of Selecting and Reporting Intraclass Correlation Coefficients for Reliability Research." J Chiropr Med **15**(2): 155-163.

Smith, S. M. and T. E. Nichols (2009). "Threshold-free cluster enhancement: addressing problems of smoothing, threshold dependence and localisation in cluster inference." Neuroimage **44**(1): 83-98.

Smith, S. M. and T. E. Nichols (2009). "Threshold-free cluster enhancement: addressing problems of smoothing, threshold dependence and localisation in cluster inference." Neuroimage **44**(1): 83-98.

**Appendix F. Wave 2: comparing rest period during the first (0-30 minutes) and second half (31-60 minutes) of the protocol**

*Statistical analysis*

A flexible factorial analysis were carried out to compare GMV during the resting period in-between task execution (0-30 minutes, rest condition) with GMV during the rest-only period following task execution (30-60 minutes, rest condition) in wave 2. The primary threshold for significance was set at *p* < .001, uncorrected; voxels surviving the primary threshold were further corrected for multiple testing with a family-wise error (FWE) rate approach at cluster level, with a threshold of *p* < .05.

*Results*

GMV was higher in the cerebellar lobule VII bilaterally, extending to the crus I, during the first half of the protocol (0-30 minutes, when the subjects were resting in-between task execution) compared with the second half of the protocol (31-60 minutes, when the subjects were only resting) (Table S6). This indicated a GMV reduction in these areas during rest after skill learning, No clusters of increased GMV after skill learning were found.

**Table S7. GMV investigation in wave 2.**

| Contrast | p FWE | Ce | T | MNI Coordinates | Structure |
| --- | --- | --- | --- | --- | --- |
| *Resting period in-between task execution (0-30 min) > rest-only period following task execution (30-60 min)* | | | | | |

| 1 | 1448 | .007 | 7.48 | 30 | -57 | -54 | R lobule VII, VIII |
| --- | --- | --- | --- | --- | --- | --- | --- |
| 2 | 1631 | .004 | 6.42 | -24 | -51 | -51 | L lobule VIII, VII |

**Abbreviations**: Ce, cluster extent; F, F-score
